# Macrophage subpopulation identity in *Drosophila* is modulated by apoptotic cell clearance and related signalling pathways

**DOI:** 10.3389/fimmu.2023.1310117

**Published:** 2024-01-12

**Authors:** Elliot C. Brooks, Martin P. Zeidler, Albert C. M. Ong, Iwan R. Evans

**Affiliations:** ^1^School of Medicine and Population Health and the Bateson Centre, University of Sheffield, Sheffield, United Kingdom; ^2^School of Biosciences and the Bateson Centre, University of Sheffield, Sheffield, United Kingdom

**Keywords:** macrophage, Drosophila, apoptotic cell clearance, haemocyte, apoptosis, ecdysone

## Abstract

In *Drosophila* blood, plasmatocytes of the haemocyte lineage represent the functional equivalent of vertebrate macrophages and have become an established *in vivo* model with which to study macrophage function and behaviour. However, the use of plasmatocytes as a macrophage model has been limited by a historical perspective that plasmatocytes represent a homogenous population of cells, in contrast to the high levels of heterogeneity of vertebrate macrophages. Recently, a number of groups have reported transcriptomic approaches which suggest the existence of plasmatocyte heterogeneity, while we identified enhancer elements that identify subpopulations of plasmatocytes which exhibit potentially pro-inflammatory behaviours, suggesting conservation of plasmatocyte heterogeneity in *Drosophila*. These plasmatocyte subpopulations exhibit enhanced responses to wounds and decreased rates of efferocytosis when compared to the overall plasmatocyte population. Interestingly, increasing the phagocytic requirement placed upon plasmatocytes is sufficient to decrease the size of these plasmatocyte subpopulations in the embryo. However, the mechanistic basis for this response was unclear. Here, we examine how plasmatocyte subpopulations are modulated by apoptotic cell clearance (efferocytosis) demands and associated signalling pathways. We show that loss of the phosphatidylserine receptor Simu prevents an increased phagocytic burden from modulating specific subpopulation cells, while blocking other apoptotic cell receptors revealed no such rescue. This suggests that Simu-dependent efferocytosis is specifically involved in determining fate of particular subpopulations. Supportive of our original finding, mutations in *amo* (the *Drosophila* homolog of *PKD2*), a calcium-permeable channel which operates downstream of Simu, phenocopy *simu* mutants. Furthermore, we show that Amo is involved in the acidification of the apoptotic cell-containing phagosomes, suggesting that this reduction in pH may be associated with macrophage reprogramming. Additionally, our results also identify Ecdysone receptor signalling, a pathway related to control of cell death during developmental transitions, as a controller of plasmatocyte subpopulation identity. Overall, these results identify fundamental pathways involved in the specification of plasmatocyte subpopulations and so further validate *Drosophila* plasmatocytes as a heterogeneous population of macrophage-like cells within this important developmental and immune model.

## Introduction

Macrophages are highly phagocytic cells of the vertebrate innate immune system, which are responsible for tissue homeostasis, fighting infection and removing apoptotic cells (1). Heterogeneity of the vertebrate macrophage is a fundamental component of the immune system, allowing these cells to respond to a variety of stimuli in a wide range of environments through differentiation into a range of tissue resident cell types and an ability to adopt various activation states, termed macrophage polarisation (2, 3). These activation states range from pro-inflammatory (M1-like) states associated with microbicidal activities and initial recruitment to wounds, to anti-inflammatory states (M2-like) that are associated with apoptotic cell clearance and the later stages of wound healing (M2-like) (4), with this spectrum of activation states regulated in response to immediate environmental challenges (5). Aberrant macrophage polarisation has been implicated in numerous chronic inflammatory conditions, such as Chronic Obstructive Pulmonary Disease (COPD) and atherosclerosis, which are associated with increased M2-like polarisation and M1-like polarisation, respectively (6–8). Though numerous experimental models have been exploited to facilitate our understanding of these fundamental processes, these often rely on *ex vivo* approaches that do not fully reproduce the temporal and spatial dynamics of *in vivo* biological systems. As such, low complexity *in vivo* models to study macrophage heterogeneity *in situ* have the potential to provide unique biological insights.

The fruit fly *Drosophila melanogaster* possesses an innate immune system comprising three lineages of haemocytes, specified via Serpent (Srp), the fly orthologue of the GATA transcription factors involved in vertebrate haematopoiesis (9). The plasmatocyte lineage is the dominant blood cell throughout normal development and represents the functional equivalent of vertebrate macrophages. Plasmatocytes mediate the same essential functions as macrophages, responding to wounds, fighting infection and removing apoptotic cells (efferocytosis) (10). Efferocytosis, has been particularly well studied in the fly, and multiple apoptotic cell receptors are broadly expressed across the total plasmatocyte population: those characterised include Simu and Draper (both CED1 family members), and Croquemort, which is homologous to the CD36 scavenger receptor expressed on human macrophages (11–15). Mutations in these receptors prevent efficient identification of dying cells, resulting in a build-up of uncleared apoptotic corpses *in vivo*, the persistence of which disrupts other plasmatocyte functions, such as migration and wound responses (16, 17). However, despite the undoubted similarities between plasmatocytes and macrophages, until recently there was limited evidence to suggest that plasmatocytes were as functionally or molecularly diverse as their vertebrate counterparts.

Recent studies into plasmatocyte behaviour *in vivo* reveal that these cells do not behave in a uniform manner. Following their dispersal across the embryo, plasmatocytes surrounding the ventral nerve cord appear to move randomly as if they are no longer migrating towards chemotactic cues. Imaging this random movement in stage 15 embryos revealed a wide range in migration speeds, suggesting some plasmatocytes may be developmentally programmed to have enhanced motility capabilities. Similarly, imaging plasmatocyte movements in the vicinity of a sterile wound indicated that some plasmatocytes exhibit a rapid and robust migratory response, while others at similar distances from the wound site fail to respond (17, 18). There is also remarkable variability in the number of apoptotic corpses phagocytosed by plasmatocytes (17–19). These data hint that the overall plasmatocyte population is made up of subpopulations, each of which exhibit distinct innate immune behaviours. Consistent with this cellular diversity, transcriptional profiling studies using single cell RNA sequencing (scRNAseq) approaches confirm the existence of molecularly-defined plasmatocyte clusters in larvae (20–23), while reporter studies indicate heterogeneity across the *Drosophila* lifecourse (18).

In addition to a diversity of activities at any one developmental stage, the behaviour of *Drosophila* plasmatocytes also changes markedly throughout the life cycle of the fly. During embryogenesis, plasmatocytes are highly migratory as they disperse around the embryo, shaping tissues and organs via deposition of extracellular matrix (24) and phagocytosis of apoptotic cells (25). By contrast, plasmatocytes are largely sessile during larval stages, adhering to the body wall where they proliferate under the control of Activin-β released from nearby neurons (26, 27). At the onset of metamorphosis, plasmatocyte behaviour changes once more, as these cells are reprogrammed to become more highly phagocytic and migratory to deal with the high levels of cell death associated with the tissue remodelling during this developmental stage (28). These alterations in plasmatocyte behaviour are influenced by the action of the steroid hormone 20-hydroxyecdysone (hereafter referred to as ecdysone), itself central to progression through these developmental stages which require significant tissue remodelling and apoptosis. Whether these changes in behaviour are linked to changes in subpopulation identity is unknown. Further examples of the importance of ecdysone in behavioural transition points is illustrated by the fact that embryonic plasmatocytes expressing a dominant-negative isoform of the ecdysone receptor (EcR) fail to mount effective responses to infection (29), while increasing ecdysone levels acting via the ecdysone receptor B1 stimulate plasmatocytes to become highly motile and phagocytic during pupation (30–32). Thus, plasmatocytes exhibit plasticity, with their behaviours changing across the lifecourse, according to the developmental stage, with the action of ecdysone implicated in contributing to these changes.

Following up *in vivo* evidence hinting at plasmatocyte heterogeneity and plasticity, we recently exploited the Vienna Tiling (VT) array library of non-coding enhancer elements (33) and identified enhancers active in a subset of plasmatocytes (18). This approach revealed the existence of functionally-distinct subpopulations that were associated with enhanced migratory responses to wounds and decreased rates of apoptotic cell clearance compared to the overall plasmatocyte population, behaviours which are typical of pro-inflammatory macrophages. These subpopulations are developmentally regulated, with relatively high numbers in embryonic stages preceding a drastic decrease in larval stages, before subpopulations ultimately re-emerge at the onset of metamorphosis and persist into adulthood (18), patterns consistent with the changes in plasmatocyte behaviour seen during development, including those regulated by ecdysone signalling.

The plasticity of *Drosophila* plasmatocytes can also be observed by increasing the apoptotic challenge they face *in vivo*. During embryogenesis, the other major cell-type involved in efferocytosis are glial cells, specified via the transcription factor *repo* (14, 34, 35). Loss of glial specification in *repo* null embryos results in increased levels of uncleared apoptotic cells – a change previously shown to impair plasmatocyte migration and wound responses (36). Interestingly, the plasmatocyte subpopulations studied to date exhibit a significant decrease in their relative numbers in a *repo* mutant background (18); this suggests that the high-apoptotic environment seen in *repo* mutants may either prevent plasmatocytes acquiring pro-inflammatory subpopulation identities or alternatively drive exit from those subpopulations.

The fact that putative pro-inflammatory macrophage subpopulations decrease in the presence of high levels of uncleared apoptotic cells suggests that signalling downstream of apoptotic cell receptors may influence subpopulation fate. Here, we show that Simu, a receptor for apoptotic cells, mediates decreases in the numbers of specific plasmatocyte subpopulation cells on exposure to enhanced levels of apoptosis. Furthermore, mutations affecting the calcium-permeable cation channel Amo, which regulates calcium homeostasis downstream of phagocytosis (37), phenocopy results seen in *simu* mutants. This is consistent with a model whereby Amo functions downstream of Simu to facilitate plasmatocyte reprograming to alternative fates. Finally, we demonstrate a requirement for ecdysone signalling in the establishment of subpopulation identity, both in the embryo and pupa. Overall, these findings further reinforce the utility of *Drosophila* plasmatocytes as a robust macrophage model – with cells clearly exhibiting heterogeneity, plasticity, and the apparent ability to switch activation states in response to different environmental challenges.

## Methods

### Fly genetics and reagents

Stocks of *Drosophila* were kept at 25°C on standard cornmeal/molasses agar. To collect embryos, at least 20 male and 20 female flies were placed in a 100mL beaker which was capped with a 5cm apple juice agar plate supplemented with a small amount of yeast paste (50% in dH_2_O), secured with an elastic band and incubated at 22°C for embryo collections. For pupal experiments, crosses were kept at 25°C and white pre-pupae were collected during 30-minute windows and aged at 25°C. All transgenes and mutations were crossed into a *w^1118^* background and *CyO dfd-nvYFP* and *TM6b dfd-nvYFP* were used as fluorescent balancers to enable genotyping of embryos (**Supplementary Table 1**).

The following *Drosophila* drivers and constructs were used: *crq-GAL4* (38), *srpHemo-GAL4* (39), *hml(Δ)-GAL4* (40), *VT17559-GAL4, VT32897-GAL4, VT57089-GAL4, VT62766-GAL4* (18, 33). When exploiting the split GAL4 system (41), the GAL4 activation domain (AD) was expressed via *srpHemo-AD*, while the GAL4 DNA binding domain (DBD) was expressed via *srpHemo-DBD*, *VT17559-DBD*, *VT32897-DBD, VT57089-DBD* and *VT62766-DBD* (18). The following UAS lines were used: *UAS-Stinger* (42), *UAS-eGFP* (Bloomington *Drosophila* Stock Centre), *UAS-drpr-II* (43), and *UAS-EcR.B1^ΔC655^* (44). The following GAL4-independent reporter lines were used: *srpHemo-3x-mCherry*, *srpHemo-H2A-3x-mCherry* (45), *srpHemo-GMA* (James Bloor, University of Kent), *VT17559-RFP, VT32897-RFP, VT57089-RFP* and *VT62766-RFP* (18). The following *Drosophila* mutants were used: *amo^1^* (46), *simu^2^* (14), *repo^03702^* (47) and *crq^ko92^* (48). **Supplementary Table 1** contains a full list of genotypes (including parental genotypes) used in this paper.

### Imaging of *Drosophila* embryos

All embryos were dechorionated in bleach (49) prior to being mounted ventral-side-up on double-sided tape (Scotch) in a minimal volume of Voltalef oil (VWR). All imaging of embryos was carried out on an UltraView Spinning Disk System (Perkin Elmer) using a 40x UplanSApo oil immersion objective lens (NA 1.3). The ventral region (most medial body segments) of embryos was imaged from the embryonic surface to a depth of 20µm, with z-slices spaced every 1µm. For lysotracker experiments requiring staining of live embryos, stage 15 dechorionated embryos were selected and transferred to a 50:50 mixture of peroxide-free heptane (VWR) and 10μM lysotracker red (Thermofisher) in PBS (Oxoid) in a glass vial, which was shaken in the dark for 30 minutes. Embryos were then transferred into a Watchmaker’s glass containing Halocarbon oil 700 (Sigma), before being mounted as described above.

Embryos requiring fixation and immunostaining were fixed and stained as previously described (17). For Fascin staining, embryos were treated with a mouse anti-Fascin primary antibody (sn7c; Developmental Studies Hybridoma Bank; used at 1:500), with Alexa fluor 568 goat anti-mouse used as a secondary antibody (A11031, Life Technologies; 1:200).

### Imaging of pupae

Pupae of the appropriate genotypes were selected and aged to 48h after puparium formation (APF) at 25°C and attached to slides via double-sided tape. Pupae were carefully removed from their pupal cases and covered in a small volume of Voltalef oil. Stacks of 5 coverslips (22 x 22mm, thickness 1) were glued together with nail varnish and then placed either side of pupae. A coverslip (22 x 32mm, thickness 1) was then placed over the top of the pupae, in contact with the oil, supported by the coverslip stacks to prevent damage to pupae. Z-stacks were then taken of the thoracic regions of pupae using a Nikon spinning disk system (Nikon Eclipse Ti2 microscope with a CSU-W1 Okagawa confocal scanner unit and Photometrics Prime 95B 22mm camera; 20X Plan Apo/0.75 objective lens, GFP and RFP filters, 2µm between slices).

### Image and statistical analyses

All images were converted to a tiff format, despeckled to reduce background noise, and blinded prior to analysis, which was performed using Fiji/ImageJ (50). To work out the relative number of plasmatocytes within subpopulations in embryos, z-stacks were converted into a maximum intensity projection in Fiji. The total number of plasmatocytes, labelled with pan-plasmatocyte reporters (such as *srpHemo-3x-mCherry*, *crq-GAL4,UAS-GFP;srpHemo-GAL4,UAS-GFP* or anti-Fascin staining), was counted. Numbers of subpopulation plasmatocytes were quantified from images of cells labelled using *VT-GAL4* lines (33), or *srpHemo-AD* in concert with *VT-DBD* transgenes (18) to drive expression from UAS reporters, or via GAL4-independent *VT-RFP* lines (18). The proportion of plasmatocytes within a given subpopulation was then expressed as a percentage of the overall population.

To quantify phagosome acidification, the number of phagosomes of the 5 most-ventral plasmatocytes were selected using the multi-point selection tool (GFP channel; phagosomes exclude cytoplasmic GFP present in plasmatocytes) from z-stacks of the ventral surface imaged as described above. These most-superficial cells are typically, but not exclusively, on the ventral midline, and the number of these phagosomes that overlapped with lysotracker red staining was counted to work out a percentage of phagosome acidification (RFP channel of the z-stacks); again the multi-point selection tool was used to ensure accuracy of scoring. All images were blinded ahead of quantification.

For quantification of subpopulations within the pupal thorax (at 48h APF), maximum projections corresponding to 10 z-slices were assembled starting from the z-position in which the most superficial plasmatocytes were visible (labelled via *hml(Δ)-GAL4,UAS-GFP*), moving deeper into the pupa. All images in the dataset underwent identical contrast adjustment and were blinded ahead of analysis. Cells were then scored as *hml*-positive only or double-positive for *hml* and *VT62766* on the basis of the presence/absence of visible fluorescence in the GFP (*hml(Δ)-GAL4,UAS-GFP*) and RFP (*VT62766-RFP*) filter channels. Cells obscured by the bounding vitelline membrane (“rings” RFP channels of pupal images) were not assessed. The multi-point tool in Fiji was used to keep track of cells that had been assessed. To normalise total numbers of *hml*-positive cells within the thoracic region, the area bounded by the vitelline membrane was measured using the polygon selection tool in Fiji. As an additional means of quantification, the same maximum projections were cropped to the region of interest (demarcated by the vitelline membrane). The green channel was then manually thresholded to create a mask corresponding to plasmatocyte localisation. This was then used to measure total fluorescence (integrated density) within *hml*-positive plasmatocytes for GFP and RFP channels to quantify reporter activity.

Statistical analysis was conducted in GraphPad Prism. Mann-Whitney and Student’s unpaired t-tests were used to compare non-parametric and parametric data, respectively. Where greater than two means were to be compared a one-way ANOVA with Dunnett’s post-test was used (parametric data).

## Results

### Loss of Simu, an apoptotic cell receptor, drives expansion of specific plasmatocyte subpopulations

**Figure 1:**
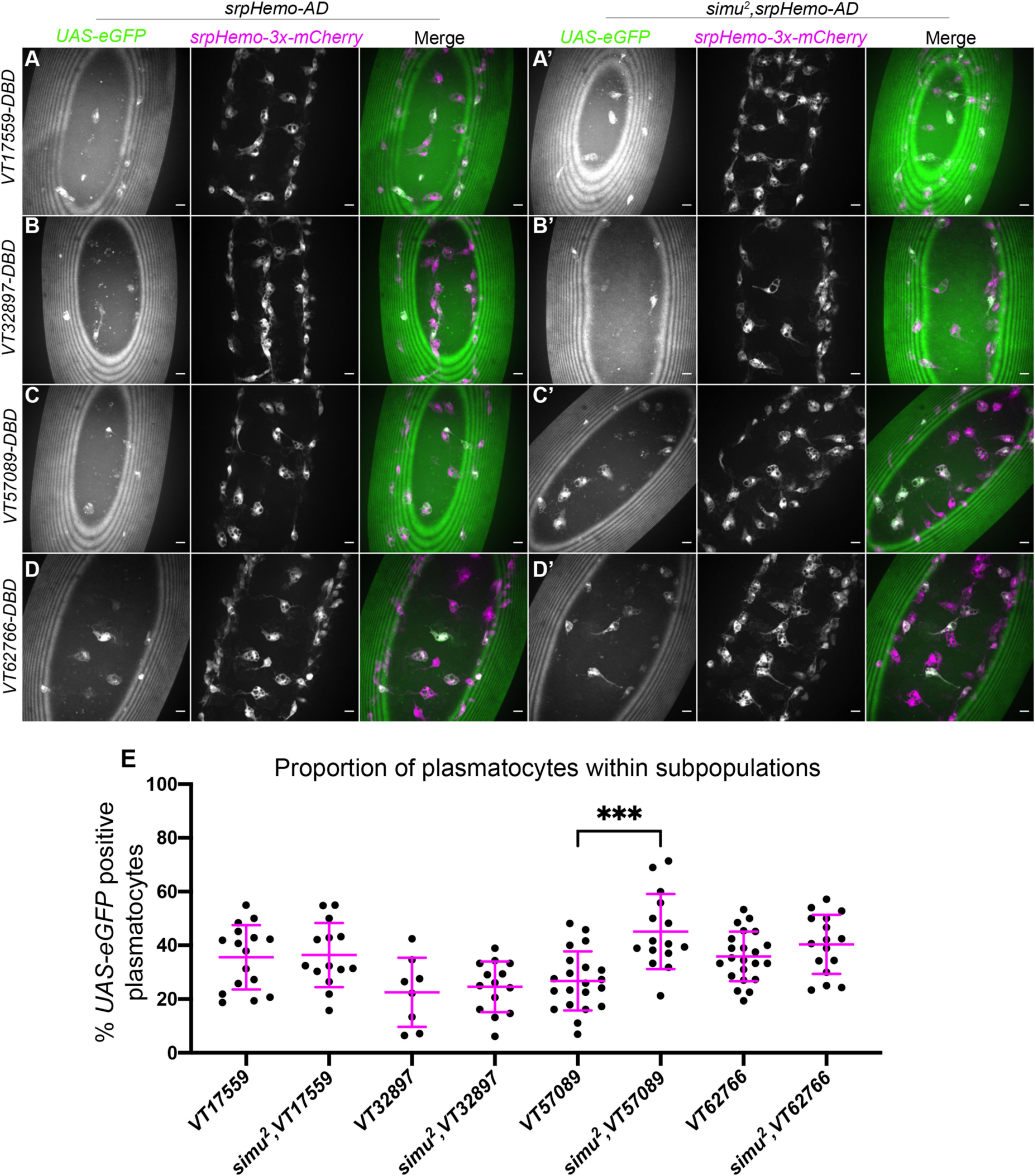
Overexposure of plasmatocytes to apoptotic cells via loss of *simu* is not sufficient to cause a decrease in plasmatocyte subpopulations. **(A-D’)** representative images of the ventral midline of control **(A–D)** and *simu^2^* **(A’-D’)** embryos at stage 15. *UAS-eGFP* shows plasmatocytes labelled via split-*GAL4* (green in merge), while *srpHemo-3x-mCherry* labels every plasmatocytes (magenta in merge). Anterior is up in all images, scale bars denote 10µm. **(E)** scatterplot showing proportion of plasmatocytes within subpopulations in control and *simu^2^* embryos. *n*=16, 14, 8,14, 21, 15, 22 and 16, respectively. Only significantly different results are shown on the graph (p=0.0003), all statistical comparisons were carried out via non-paired t-tests. *** Denotes p<0.001.

We previously described how numbers of haemocytes in subpopulations defined by the *VT17559*, *VT32897*, *VT57089* and *VT62766* enhancers are reduced in a genetic background containing excess apoptotic cells (*repo* mutants; 18, 36). To explore the mechanistic basis for this effect, we examined the effect of loss of *simu* upon macrophage subpopulations in stage 15 embryos (**Figure 1**). Subpopulation plasmatocytes were labelled via the split GAL4 system (41) driving *UAS-GFP* specifically in cells with overlapping expression of *serpent* (*srpHemo-AD*) and the subtype-specific VT enhancer (*VT-DBD*) activity (18), while the total plasmatocyte population was independently labelled via a GAL4-independent reporter (*srpHemo-3x-mCherry*; 45; labelled in magenta in **Figure 1**).

In contrast to *repo* mutants, where an increased apoptotic challenge correlates with a decrease in numbers of subpopulation plasmatocytes (18), no change was observed for the *VT17559*, *VT32897* and *VT62766* subpopulations in a *simu* mutant background (**Figures 1A–E**), while numbers of *VT57089*-labelled cells actually increased in *simu* mutants compared to controls (**Figures 1C, E**). This suggests that Simu normally antagonises acquisition or maintenance of the *VT57089* fate. These results suggest that the relative decreases in subpopulation cells seen in *repo* mutants (18) may depend upon the presence of Simu on the surface of plasmatocytes.

### Simu mediates antagonism of specific subpopulation fates in the presence of large amounts of apoptosis

**Figure 2:**
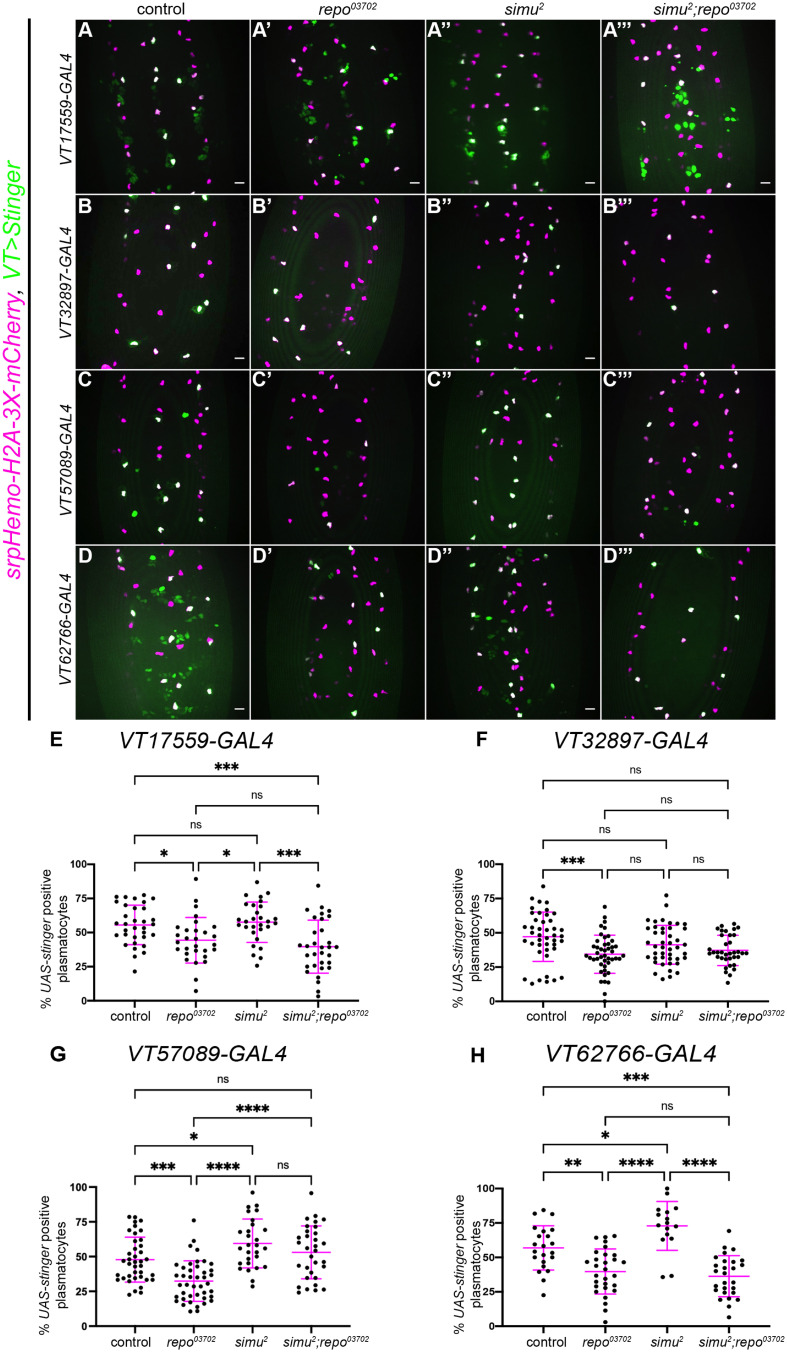
Simu-dependent efferocytosis is required for the decrease in the *VT57089* subpopulation seen in *repo* mutants. **(A-D’’’)** representative maximum projection images of the ventral midline of control **(A–D)**, *repo^03702^* single mutants **(A’-D’)**, *simu^2^* single mutant **(A’’’-D’’’)** and *simu^2^;repo^03702^* double mutant **(A’”-D’”)** embryos at stage 15. *UAS-Stinger* shows subpopulation plasmatocytes labelled via *VT*-*GAL4* (green in merge), while *srpHemo-H2A-3x-mCherry* labels every plasmatocytes (magenta in merge). Anterior is up in all images, scale bars denote 10µm. **(E)** scatterplot showing proportion of plasmatocytes within the *VT17559* subpopulation. *n*=33, 29, 29 and 32, respectively. **(F)** scatterplot showing proportion of plasmatocytes within the *VT32897* subpopulation. *n*=44, 45, 43 and 36, respectively. **(G)** scatterplot showing proportion of plasmatocytes within the *VT57089* subpopulation. *n*=39, 42, 28 and 32, respectively. **(H)** scatterplot showing proportion of plasmatocytes within the *VT62766* subpopulation. *n*=22, 29, 16 and 27, respectively. Statistical analyses carried out via one-way ANOVA with Dunnett’s multiple comparison test. *, **, *** and **** represent p<0.05, p<0.01, p<0.001 and p<0.0001, respectively.

The contrasting results seen between *repo* and *simu* mutant embryos indicate that increased levels of uncleared apoptotic cells may not be sufficient to mediate decreases in subpopulation numbers – instead, specific interactions between apoptotic cells and plasmatocytes may be required for phenotypic switches. To test whether effective recognition and/or engulfment of apoptotic cells is responsible for mediating the reduction in subpopulation plasmatocytes observed in *repo* mutants, *simu;repo* double mutants were generated and compared to controls (as well as *simu* and *repo* single mutants). Due to the genetics involved in this experiment, subpopulation plasmatocytes were labelled via *VT-GAL4* (as opposed to split *VT-GAL4*) driving expression of *UAS-Stinger* (**Figures 2A–D**).

As we have previously shown (18), fewer plasmatocytes were present in all subpopulations examined in the presence of *repo* mutations alone (**Figures 2E–H**). The loss of *simu* in addition to *repo* (*simu;repo* double mutants) was unable to rescue relative plasmatocyte subpopulation numbers to control levels for the *VT17559* and *VT62766* reporters (**Figures 2E, H**); there is considerable variability in subpopulation numbers and results for the *VT32897* reporter were not statistically significant (**Figure 2F**), though the trends were consistent with those observed for *VT17559* and *VT62766* (**Figures 2E, H**). This variability potentially stems, in part, from the stochastic nature of contact between apoptotic corpses and plasmatocytes in the embryo. In contrast, numbers of cells labelled via the *VT57089* reporter were completely rescued to control levels in *simu;repo* double mutants (**Figure 2G**). This implies that Simu-dependent efferocytosis, or an effector downstream of Simu-dependent recognition of apoptotic cells, may mediate the apparent shift out of the *VT57089* subpopulation seen in the presence of large numbers of apoptotic cells. Furthermore, this suggests distinct mechanisms may control plasmatocyte identity from subpopulation to subpopulation in response to the high apoptotic challenge presented to plasmatocytes (as assayed via *repo* loss-of-function).

### Other apoptotic cell clearance receptors do not appear to contribute to subpopulation identity

**Figure 3:**
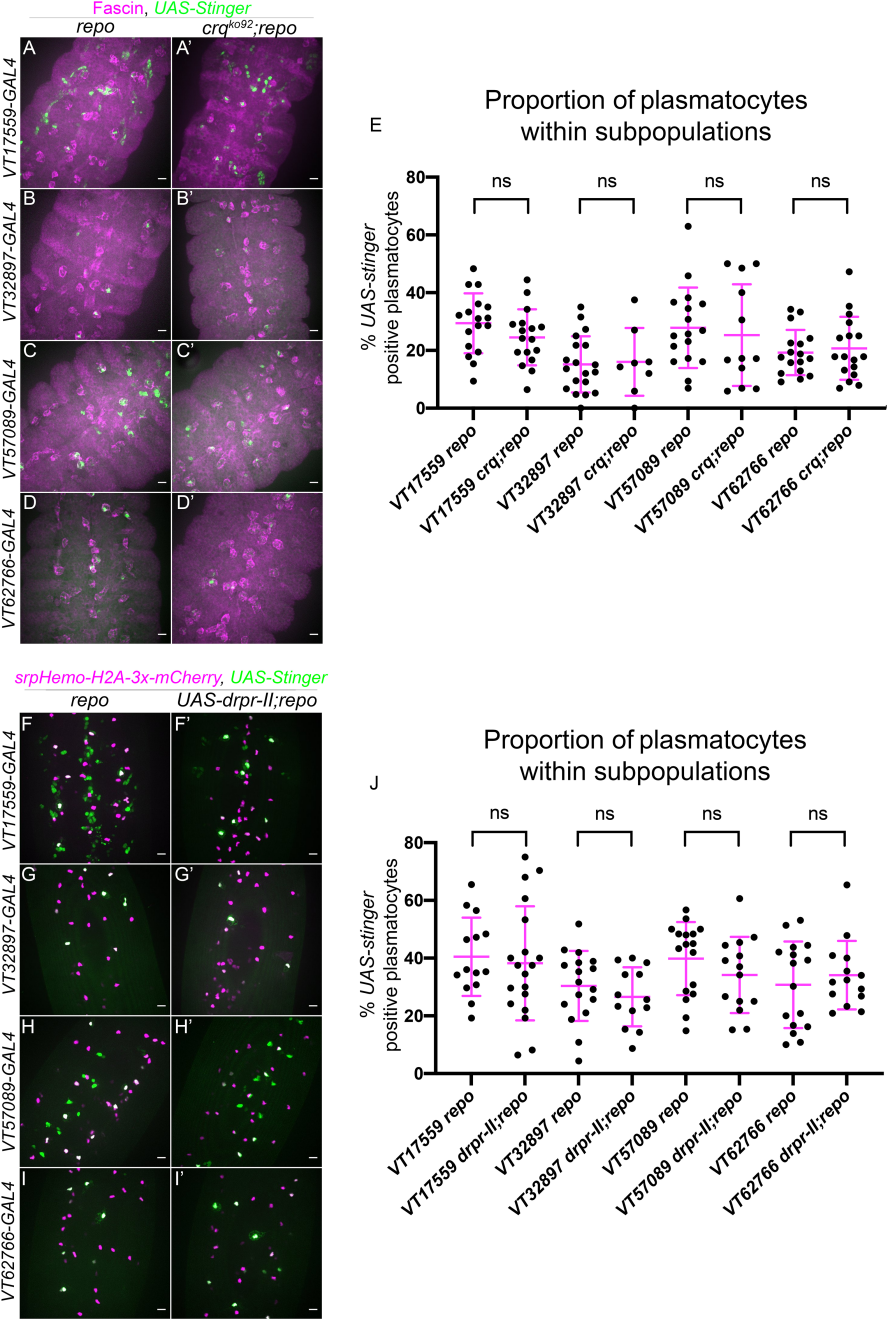
Croquemort and Draper do not affect plasmatocyte subpopulations. **(A-D’)** representative maximum projection images of the ventral midline of *repo*-only single mutant embryos **(A–D)** and *crq;repo* double mutant embryos **(A’-D’)** at stage 15. Plasmatocytes labelled via Fascin staining (magenta) while subpopulation plasmatocytes are labelled via *VT-GAL4* driving expression of *UAS-Stinger* (green). Anterior is up in all images, scale bars denote 10µm. **(E)** scatterplot showing proportion of plasmatocytes within subpopulations. *n=* 18, 17, 20, 8, 17, 12, 17 and 17, respectively. Statistical analyses carried out via unpaired *t*-tests. **(F-I’)** representative maximum projection images of the ventral midline of *repo*-only single mutant embryos **(F–I)** and *repo* mutant embryos expressing *UAS-drpr-II* specifically in plasmatocytes **(F’-I’)** at stage 15. All plasmatocytes labelled via srpHemo-H2A-3x-mCherry, while subpopulation plasmatocytes are labelled via VT-GAL4 lines driving expression from UAS-Stinger. Anterior is up in all images, scale bars denote 10µm. **(J)** scatterplot showing proportion of plasmatocytes within subpopulations. *n*=14, 19, 17, 13, 17, 14, 16 and 14, respectively. Statistical analyses in **(E, J)** carried out via unpaired *t*-tests.

Given the role of *simu*, we next examined the role of *crq* and *drpr* in regulation of subpopulation identity following challenge with large numbers of apoptotic cells (i.e., in a *repo* mutant background). A loss-of-function *crq* allele (*crq^ko^*; 48) was used alongside *repo* mutations to investigate whether *crq* is required for the decreased numbers of subpopulation plasmatocytes seen in *repo* mutants. Subpopulation plasmatocytes were labelled via *VT-GAL4* transgenes driving *UAS-Stinger*, while all plasmatocytes were labelled via immunostaining for Fascin, an Actin-bundling protein highly enriched in *Drosophila* plasmatocytes (51; **Figures 3A–D**). This approach revealed no differences in the proportion of plasmatocytes found within each subpopulation when comparing *repo* single mutants to *crq;repo* double mutants (**Figure 3E**). This suggests that the decrease in subpopulation numbers seen in *repo* mutants is independent of Crq.

To investigate the involvement of *drpr* in modulating plasmatocyte subpopulations, an inhibitory isoform of *drpr* (*UAS-drpr-II*; 43) was expressed specifically in subpopulation plasmatocytes. This was driven using *VT-GAL4* transgenes, which simultaneously enabled expression of *UAS-Stinger* to label subpopulation cells; the overall plasmatocyte population was labelled via *srpHemo-H2A-3x-mCherry* (**Figures 3F–I**). Similar to the use of *crq* mutants, expression of *drpr*-II specifically in subpopulation cells in a *repo* mutant background did not impact subpopulation numbers when compared to *repo* mutants lacking *UAS-drpr-II* expression (**Figures 3F–J**). Consistently, expression of *drpr-II* in all plasmatocytes did not impact subpopulation numbers in the absence of increased apoptotic cell challenge (**Supplementary Figures 1**, **2**). Taken together this suggests that neither Drpr nor Crq modulate subpopulation identity at embryonic stages, in contrast to Simu.

### Amo functions downstream of Simu to control identity of specific subpopulations

**Figure 4:**
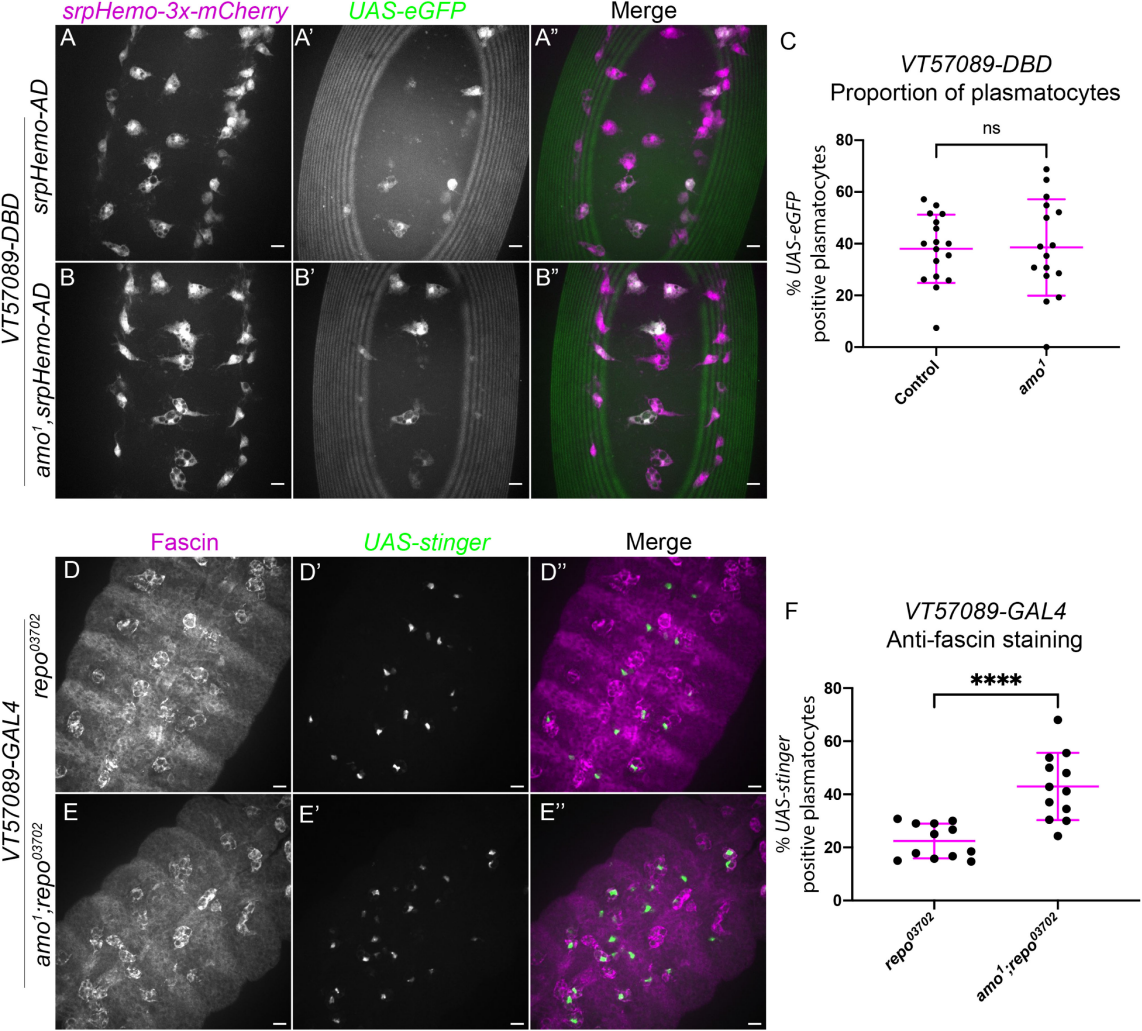
The calcium-permeable cation channel Amo is required for the shift out of the *VT57089* subpopulation seen in *repo* mutants. **(A-B”)** representative images of the ventral midline of control **(A-A”)** and *amo^1^* **(B-B”)** embryos at stage 15. *srpHemo-3x-mCherry* labels every plasmatocyte (magenta in merge), while *VT57089* subpopulation plasmatocytes labelled via split *GAL4* system to drive expression of *UAS-eGFP* (green in merge). Anterior is up in all images, scale bars denote 10µm. **(C)** scatterplot showing the proportion of plasmatocytes within the *VT57089* subpopulation in control and *amo^1^* embryos. *n*=17 and 16, respectively. **(D-E”)** representative maximum projection images of the ventral midline of *repo^03702^* single mutant **(D-D”)** and *amo^1^;repo^03702^* double mutant **(E-E”)** embryos at stage 15. Plasmatocytes have been labelled via Fascin staining (magenta in merge), with *VT57089* positive cells labelled via *UAS-Stinger* (green in merge). Anterior is up in all images, scale bars denote 10µm. **(F)** scatterplot showing proportion of plasmatocytes within the *VT57089* subpopulation between *repo^03702^* single mutant and *amo^1^;repo^03702^* double mutant embryos based on anti-Fascin staining. *n*=12 and 13, respectively. Statistical analyses in **(C, F)** carried out via unpaired *t*-tests. **** represents p<0.0001.

Our results so far have shown that Simu appears to be involved in shifting plasmatocytes out of the *VT57089* subpopulation, both in control backgrounds (**Figure 1E**) and in response to the high apoptotic challenge presented by *repo* mutations (**Figure 2G**). The calcium-permeable cation channel Amo, homologous to human *PKD2*, which is causative of Autosomal Dominant Polycystic Kidney Disease (ADPKD), has previously been shown to maintain calcium homeostasis downstream of Simu during later stages of efferocytosis in *Drosophila* (37). We therefore used a loss-of-function *amo* allele (*amo^1^*; 46) to investigate whether Amo-dependent calcium homeostasis is involved in modulating the identity of *VT57089* subpopulation plasmatocytes in stage 15 embryos. As per **Figure 1C**, *VT57089* subpopulation plasmatocytes were labelled using the split GAL4 system to drive expression of *UAS-eGFP*, while the overall plasmatocyte population was labelled via *srpHemo-3x-mCherry* (**Figures 4A, B**).

Unlike *simu* mutants, no differences in the proportion of plasmatocytes within the *VT57089* subpopulation were observed when comparing wild-type embryos to *amo^1^* single mutants (**Figure 4C**). These experiments were initially conducted in embryos with ‘normal’ – i.e., developmental levels – of apoptosis. We therefore next introduced *repo* mutations to address how loss of Amo impacted the *VT57089* subpopulation in response to an increased apoptotic challenge. Subpopulation plasmatocytes were labelled using *VT-GAL4* transgenes to drive expression from *UAS-Stinger*, while anti-Fascin staining was again used to label the overall macrophage population to calculate the proportion of plasmatocytes within this subpopulation (**Figures 4D–E**). Interestingly, these results phenocopied *simu* mutants, with a significantly higher proportion of *VT57089* plasmatocytes found in *amo;repo* double mutants compared to *repo*-only controls (**Figure 4F**). This suggests that Amo-mediated calcium homeostasis is an important aspect of signalling downstream of Simu, which may prevent acquisition of *VT57089* identity, or mediate reprogramming of plasmatocytes away from this specific subpopulation.

**Figure 5:**
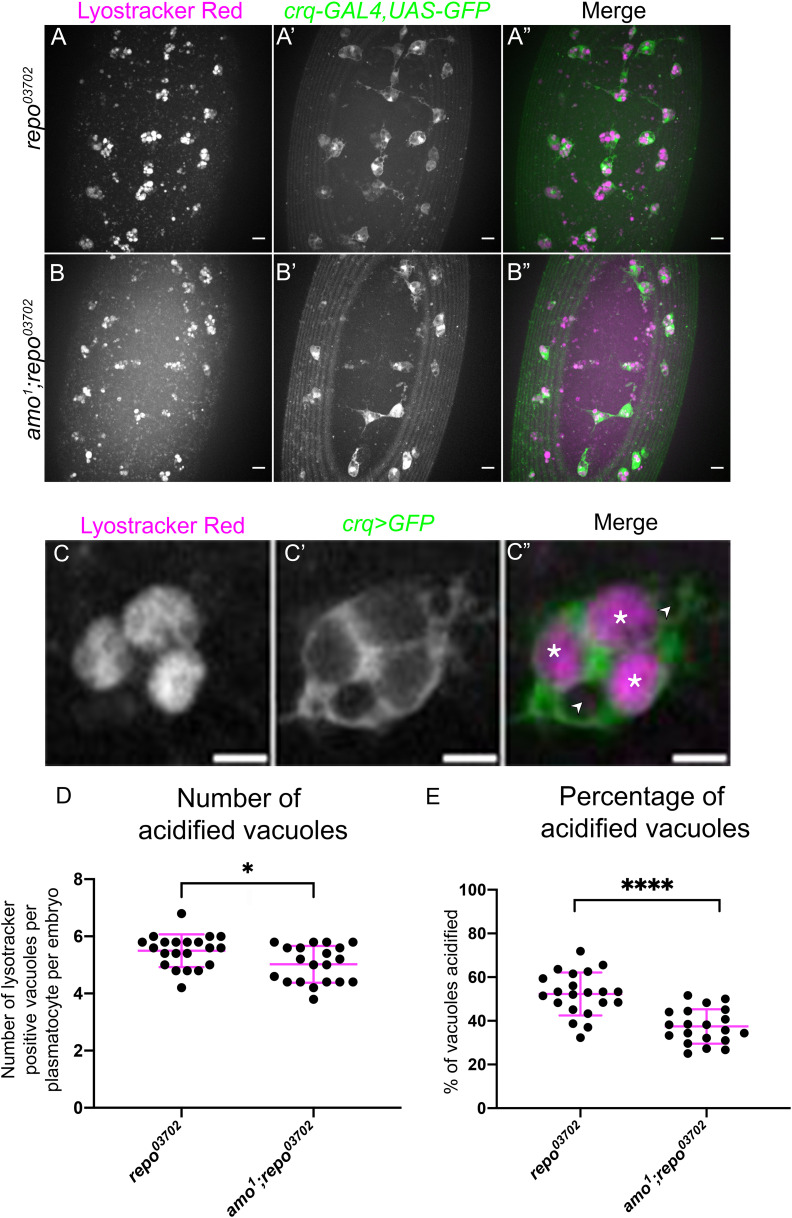
Amo is required for effective phagosome acidification. **(A-B”)** representative maximum projection images of the ventral midline of *repo^03702^* single mutant **(A-A”)** and *amo^1^;repo^03702^* double mutant **(B-B”)** embryos at stage 15. Lysotracker red shows acidified phagosomes **(A, B)**, while all plasmatocytes are labelled via *crq-GAL4,UAS-eGFP* **(A’-B’)**. Anterior is up in all images, scale bars denote 10µm. **(C-C”)** zoom of a representative plasmatocyte showing both acidified (marked with an asterisk) and non-acidified (marked with an arrowhead) phagosomes. Scale bar denotes 5µm. **(D)** scatterplot showing average number of lysotracker red positive vacuoles per plasmatocyte. *n*=21 and 20, respectively. Statistical analysis carried out via Mann-Whitney test; * represents p<0.05. **(E)** scatterplot showing proportion of vacuoles counted which were lysotracker red positive (i.e., acidified). *n*=21 and 20, respectively. Statistical analyses carried out via unpaired *t*-tests; **** represents p<0.0001.

To investigate the functional impact of *amo* mutations in the presence of elevated apoptotic challenge – i.e., in a *repo* mutant background – lysotracker staining was utilised to visualise defects in phagosome acidification. Phagosome acidification occurs during the later stages of efferocytosis, downstream of engulfment (52), and is a calcium-dependent process (53). Thus, this process was an attractive pathway to investigate with respect to *amo* mutations, due to the calcium permeability of the Amo cation channel. All plasmatocytes were labelled via *crq-GAL4* driving expression of *UAS-eGFP* (**Figures 5A–C**), and the number of acidified (lysotracker red positive) vacuoles per plasmatocyte was quantified. Lysotracker staining of *repo* single mutants and *amo;repo* double mutants revealed a significant reduction in the total number of acidified phagosomes present within in *amo*;*repo* double mutant plasmatocytes compared to *repo*-only controls (**Figure 5D**). Furthermore, the proportion of phagosomes that were acidified was also significantly lower in *amo;repo* double mutants (**Figure 5E**). These results suggest that Amo is either required for phagosomal acidification or plays an upstream role in that process during efferocytosis. Defects in acidification may interfere with specification of plasmatocytes as *VT57089* subpopulation cells. Alternatively, this process could lead to plasmatocytes exiting this subpopulation fate. Overall, we have shown that Simu negatively regulates *Drosophila* plasmatocyte subpopulation identity. *Amo* also appears required, with its role potentially linking effective phagosome acidification during the later stages of efferocytosis to regulation of cell identity.

### Ecdysone signalling contributes to regulation of subpopulation identity

**Figure 6:**
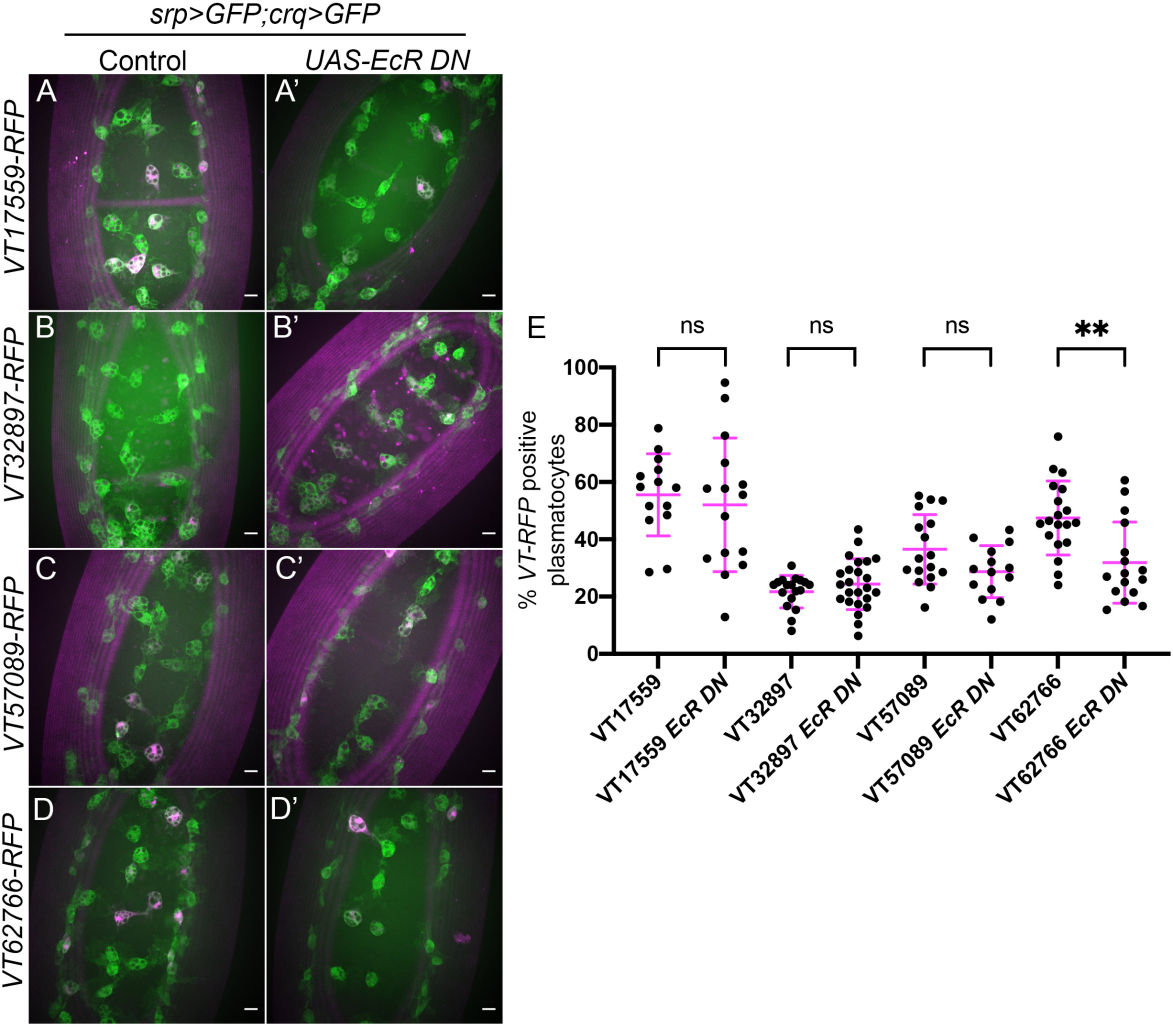
Ecdysone is required for establishing the *VT62766* subpopulation in the embryo**. (A-D’)** representative maximum projection images of the ventral midline of control embryos **(A–D)** and embryos expressing *UAS-EcR^ΔC655^* (*EcR DN*) specifically in plasmatocytes **(A’-D’)** at stage 15. Plasmatocytes labelled via *srpHemo-GAL4,UAS-GFP* and *crq-GAL4,UAS-GFP* (green) while subpopulation plasmatocytes are labelled via *VT-RFP* transgenes (magenta). Anterior is up in all images, scale bars denote 10µm. **(E)** Scatterplot showing proportions of plasmatocytes within subpopulations in the presence and absence of pan- plasmatocyte *UAS-EcR^ΔC655^* expression. *n*=14, 15, 18, 24, 17, 14, 19 and 16, respectively. Statistical analyses carried out via unpaired t-tests, ** represents p<.0.01.

In the context of *Drosophila* development and metamorphosis, the levels of the steroid hormone ecdysone (20-hydroxyecdysone) are absolutely central to a multitude of developmental events (54) – including the switching of plasmatocytes between different behavioural and transcriptional states (28, 31). Given these important roles in programming of *Drosophila* immune cells and association of ecdysone with developmental transitions during which there are significant changes in plasmatocyte subpopulation numbers, we sought to investigate whether this hormone also plays an instructive role in establishing subpopulation identity. A dominant-negative isoform of the nuclear ecdysone receptor (*UAS-EcR.B1^ΔC655^;* referred to hereafter as *EcR-DN*; 44) was therefore specifically expressed in all plasmatocytes via *srpHemo-GAL4* and *crq-GAL4*, with subpopulations labelled via GAL4*-*independent *VT-RFP* reporters, with the percentage of plasmatocytes within each subpopulation then determined (**Figures 6A–D**).

The proportion of plasmatocytes within the *VT17559* and *VT32897* subpopulations was unchanged on expression of EcR-DN, suggesting that ecdysone signalling is not responsible for establishing these subpopulations in the embryo (**Figure 6E**). Although the *VT57089* subpopulation exhibited a trend suggesting a modest decrease in numbers in the presence of EcR-DN, this was not statistically significant (**Figure 6E**). By contrast, the *VT62766* subpopulation exhibited a 30% decrease of subpopulation plasmatocytes in the presence of EcR-DN, suggesting ecdysone signalling is autonomously required in plasmatocytes for the differentiation and/or maintenance of this subpopulation in the embryo (**Figure 6E**).

**Figure 7:**
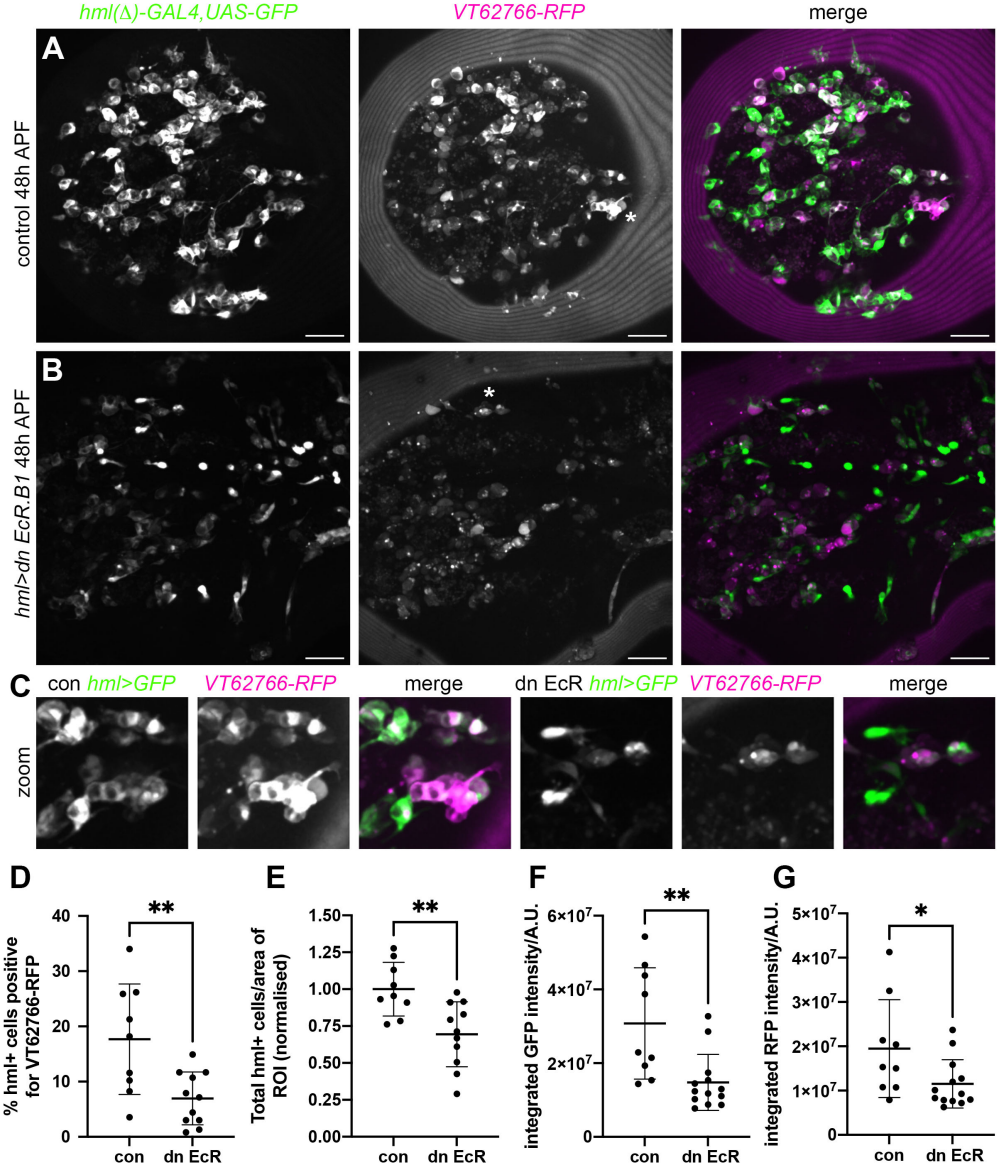
Ecdysone signalling regulates *VT62766* subpopulation cells in pupae. **(A, B)** Maximum projections of thoracic regions at 48h APF of a control pupa **(A)** and a pupa in which dominant negative EcR.B1 (*hml>dn EcR.B1*), **(B)** was expressed in haemocytes via *hml(Δ)-GAL4*. Pupae contain *hml(Δ)-GAL4,UAS-GFP* (*hml>GFP*, green in merge) and *VT62766-RFP* (magenta in merge) to label haemocytes and *VT62766*-expressing cells, respectively. **(C)** zooms of asterisked areas in **(A, B)**, which contain examples of haemocytes positive for both GFP and RFP. Scale bars denote 50µm **(A, B)**. **(D–G)** Scatterplots showing percentage of *hml*-positive cells that also express *VT62766-RFP* **(D)**, total number of *hml*-positive cells per area of the thorax analysed (normalised according to the control mean) **(E)**, and quantification of GFP and RFP levels within cells segmented as *hml*-positive (integrated GFP and RFP fluorescent intensities under a GFP mask; **F, G**). Lines and error bars represent mean and standard deviation; * and ** denote p<0.05 and p<0.01, respectively, following Student’s t-tests **(D, E)** or Mann-Whitney test (**F, G**).

*VT62766*-labelled cells appear absent during late larval stages but can once more be found in large numbers in pupae (18). Haemocytes are exposed to multiple waves of ecdysone during pupal development. Therefore, to test whether ecdysone signalling contributed to re-emergence of this population of cells, we manipulated ecdysone signalling specifically within haemocytes in pupae. As in the embryo, blocking ecdysone signalling within the majority of plasmatocytes (using *hml(Δ)-GAL4* to drive expression from *UAS-GFP* and *UAS-EcR-DN*) decreased the numbers of cells that could be labelled via the *VT62766-RFP* reporter (**Figure 7**). Clear morphological differences were also obvious comparing plasmatocytes in the thorax in controls and upon overexpression of EcR-DN, whereby cells were less vacuolated and less spherical in the latter (**Figures 7A–C**).

Overall, our data provide further evidence that the immune system of *Drosophila* comprises of heterogeneous subpopulations of plasmatocytes, akin to vertebrate macrophages. The identities of plasmatocyte subpopulations appear to be modulated by distinct processes, and we have identified signalling pathways (Simu and ecdysone signalling) involved in the establishment of specific subpopulations.

## Discussion

We and others have previously demonstrated macrophage heterogeneity in *Drosophila* (18, 20–23). In this study we investigated mechanisms regulating *Drosophila* macrophage subpopulations *in vivo*, focusing on signalling pathways associated with ingestion of apoptotic cells and developmental transitions and using variations in subpopulation numbers induced by apoptotic cell challenge and developmental stage as experimental tools (18). We show that the phosphatidylserine receptor Simu antagonises *VT57089* subpopulation fate and is necessary for reprogramming events that reduce numbers of this subpopulation in the face of excess apoptotic cells. Consistent with a role downstream of Simu, the calcium-permeable cation channel Amo also regulated this subpopulation. Amo was implicated in mediating effective phagosome acidification, which may represent an important process in modulating numbers of cells within the *VT57089* subpopulation. Interestingly, Simu-dependent efferocytosis did not affect other subpopulation identities, while other apoptotic cell receptors (Crq and Drpr) did not seem to integrate apoptotic cell sensing and reprogramming of immune cells. Finally, we show that ecdysone signalling, itself associated with developmental timepoints featuring significant alternations in subpopulation numbers, also impacts identity of specific subpopulations. Taken together, individual plasmatocyte subpopulations are regulated by distinct processes at specific developmental stages. These results further reinforce the model that plasmatocytes exist as a heterogeneous population of cells that are programmed (and/or re-programmed) in response to the precise *in vivo* microenvironment.

*Repo* mutants have previously been used in genetic approaches to challenge plasmatocytes with large amounts of apoptosis (18, 19, 36). Loss of *repo* prevents the specification of glia, another important phagocyte lineage in the embryo (47). This results in increased numbers of uncleared apoptotic cells, as fewer phagocytes remain to clear dying cells (15). The resulting challenge impairs effective migration and wound responses (36), and alters subpopulation numbers (18). Plasmatocytes within *repo* mutant embryos maintain expression of pan-plasmatocyte reporters (e.g., *srpHemo-GAL4*, *crq-GAL4, pxn-GAL4*) and are able to efficiently phagocytose apoptotic cells (36). Nonetheless, we cannot exclude the possibility that glia can also influence plasmatocyte specification independently of apoptosis.

Similarly, we cannot completely exclude that altered developmental dispersal accounts for differences in the number of subpopulation cells on the ventral midline (where we assay proportions of different subpopulations) upon manipulation of apoptotic cell clearance signalling pathways. However, we regard this explanation as unlikely, since plasmatocyte responses to apoptotic cell death appear highly local in the *Drosophila* embryo (17, 55) and changes in the numbers of apoptotic cells should only occur proximally to Repo-positive glia, which are absent in other regions of the embryo (e.g., along their dorsal migration route). Furthermore, if specific subpopulation cells are re-routed elsewhere in *repo* mutant embryos, it might be expected that there are differences in the total number of plasmatocytes on the ventral surface of the embryo; we do not detect differences in the total number of cells across the genotypes analysed, nor is there an increase observed between *repo* and *simu;repo* mutants where loss of *simu* rescues *repo* phenotypes (*VT57089*-labelled cells; data not shown).

Here we demonstrate a role via which Simu can antagonise acquisition of specific subpopulation identities. In both *repo* and *simu* mutant embryos, plasmatocytes face elevated levels of apoptosis (17, 36). However, increasing the levels of apoptotic cells triggered by loss of *repo* has a broad effect on the expression of multiple subpopulation reporters, whereas loss of *simu* is more specific. This suggests that, in some circumstances, contact with apoptotic cells may not be sufficient for changes in subpopulation identity. That loss of *simu* function can block *repo*-induced changes in reporter expression suggests that signalling through this apoptotic cell clearance receptor is mediating these alterations in plasmatocyte identity. Consistent with a role for Simu-dependent signalling, a cation channel known to operate downstream of Simu, Amo (37), also impacts subpopulation numbers. However, the precise mechanisms remain to be determined, not least since Simu lacks an intracellular signalling domain (14). In comparison to *simu*, *amo* expression remains less well characterised; *simu* is broadly expressed but without correlation with subpopulation identity (18). Thus, we cannot rule out that varying levels of *amo* expression contribute to differences in how individual subpopulations are controlled.

The human homolog of *amo* is *PKD2*, which encodes Polycystin-2 (PC-2). Mutations in *PKD2* and *PKD1* cause autosomal dominant polycystic kidney disease (ADPKD), a relatively common genetic nephropathy, wherein tubular epithelial cells proliferate to form cysts, ultimately resulting in renal failure (56). Though the exact mechanisms involved in the pathogenesis of ADPKD remain poorly understood, there appears to be a role for immune cells in cyst expansion, with macrophage polarisation also implicated. Monocyte chemoattractant protein (MCP-1) and macrophage migration inhibitory factor (MIF) lead to an initial influx of pro-inflammatory macrophages, which then polarise towards a more anti-inflammatory, pro-proliferative activation state, driving cyst expansion and disease progression (57–59). Our data revealed decreased phagosome acidification in *amo;repo* mutant plasmatocytes compared to *repo*-only controls. The changes in the type of innate immune cells present in ADPKD and in the fly embryo on loss of *PKD2*/*amo* suggests manipulation of phagosome maturation may represent a novel pathway to target with respect to further understanding pathogenesis of the disease.

Amo is a cation channel and calcium signals have long been linked to phagocytic events, albeit the data has not always proven consistent (53). A recent paper beautifully delineates a requirement of calcium nanodomains for activation of dynamin during phagocytosis (60), though the key channels here were NAADP-regulated two-pore channels. Phagocytosis is also associated with more global elevations in cytoplasmic calcium and these authors speculate that these may regulate changes in mitochondrial energetics and gene expression. It is plausible that Amo might contribute to global Ca^2+^ signals under conditions of phagocytic stress. In turn, this may facilitate a contribution to changes in gene expression necessary to reprogramme macrophage subpopulations. Notably, calcium signalling is already known to be associated with changes in gene expression following apoptotic cell clearance in *Drosophila* (61).

The steroid hormone ecdysone is known to be involved in mediating large-scale phenotypic changes in the overall plasmatocyte population associated with different developmental stages (28–31). For example, larval plasmatocytes, which are typically sessile and proliferative, become highly migratory and phagocytic at the onset of metamorphosis in response to ecdysone (28). Similarly, embryonic plasmatocytes are unable to mount an effective immune response until being exposed to ecdysone at stage 12 (29). Expression of a dominant-negative ecdysone receptor isoform revealed a decreased proportion of plasmatocytes within the *VT62766* subpopulation in both embryos and pupae. It is therefore possible that ecdysone is required to help establish this plasmatocyte subpopulation. Other subpopulations were not affected by this approach, highlighting specificity in how subpopulation identity is established and controlled.

Ecdysone signalling has been shown to drive expression of phagocytic genes in pupae (28), stimulate motility and cytoplasmic rearrangements (31, 62), and help establish immune responses (29). The *VT62766* subpopulation exhibits enhanced wound responses, but reduced rates of phagocytosis (18), so while ecdysone could be argued to drive cells towards a more activated state, associated changes in behaviour do not completely align. However, it is important to note that earlier papers quantify behaviour across the total population of plasmatocytes so subpopulation-specific effects could be obscured.

Like steroid hormone signalling, apoptotic cell death is also associated with reprogramming of vertebrate macrophages (4). Pro-inflammatory cytokines are inhibited in macrophages following phagocytosis of apoptotic cells (63). Contact with large numbers of apoptotic cells reprograms plasmatocytes away from identities that exhibit less efficient apoptotic cell clearance (18), which potentially might signify a less pro-inflammatory state. Loss of an apoptotic cell clearance receptor blocks that effect for at least one discrete population of cells, reinforcing differences between the cells marked using our transgenic reporters.

In summary, we have identified new molecular players involved in determining the acquisition of specific plasmatocyte subpopulation identities in *Drosophila*: Simu and the downstream effector Amo regulate *VT57089* identity, with phagosome acidification a potential point of integration. Meanwhile, ecdysone appears important in establishing identity of *VT62766*-labelled cells in both the embryo and pupa. The role of steroid hormone signalling and apoptosis suggest that mechanisms controlling innate immune cell behaviour in *Drosophila* and vertebrates may be more similar than previously thought. Finally, this further supports the existence of macrophage heterogeneity within this important immune model and will enable use of the fly to further dissect regulation of this important facet of biology *in vivo*.

## Data availability statement

The original contributions presented in the study are included in the article/**Supplementary Materials**, further inquiries can be directed to the corresponding author/s.

## Ethics statement

The manuscript presents research on animals that do not require ethical approval for their study.

## Author contributions

EB: Conceptualization, Data curation, Formal analysis, Investigation, Methodology, Validation, Visualization, Writing – original draft, Writing – review & editing. MZ: Conceptualization, Formal analysis, Methodology, Resources, Supervision, Validation, Writing – review & editing. AO: Conceptualization, Funding acquisition, Methodology, Project administration, Supervision, Writing – review & editing. IE: Conceptualization, Data curation, Formal analysis, Funding acquisition, Investigation, Methodology, Project administration, Resources, Software, Supervision, Validation, Visualization, Writing – original draft, Writing – review & editing.

## Supplementary material

Supplementary Figure 1 Pan-macrophage expression of Draper-II causes no effect on the *VT17559* or *VT32897* subpopulations. **(A-B”, D-E”)** representative maximum projection images of the ventral midline of wild-type control embryos **(A-A”, D-D”)** and embryos expressing *UAS-drpr-II* specifically in plasmatocytes **(B-B”, E-E”)** at stage 15. Plasmatocytes labelled via *srp-GAL4,UAS-GFP;crq-GAL4,UAS-GFP* **(A, B, D, E)** while subpopulation macrophages labelled via *VT17559-RFP* **(A’, B’)** or *VT32897-RFP* **(D’, E’)**. Anterior is up in all images; scale bars denote 10μm. **(C, F)** scatterplots showing proportion of plasmatocytes within the *VT17559* **(C)** and *VT32897* **(F)** subpopulations. **(C)** *n=* 20 and 22, respectively; **(F)** *n=* 19 and 17, respectively. Statistical analyses carried out via unpaired *t*-tests; ns denotes not significant; lines and error bars represent mean and standard deviation, respectively.

Supplementary Figure 2 Pan-macrophage expression of Draper-II causes no effect on the *VT57089* or *VT62766* subpopulations. **(A-B”, D-E”)** representative maximum projection images of the ventral midline of wild-type control embryos **(A-A”, D-D”)** and embryos expressing *UAS-drpr-II* specifically in plasmatocytes **(B-B”, E-E”)** at stage 15. Plasmatocytes labelled via *srp-GAL4,UAS-GFP;crq-GAL4,UAS-GFP* **(A, B, D, E)** while subpopulation macrophages labelled via *VT57089-RFP* **(A’, B’)** or *VT62766-RFP* **(D’, E’)**. Anterior is up in all images; scale bars denote 10μm. **(C, F)** scatterplots showing proportion of plasmatocytes within the *VT57089* **(C)** and *VT62766* **(F)** subpopulations. **(C)** *n=* 18 and 18, respectively; **(F)** *n=* 15 and 22, respectively. Statistical analyses carried out via unpaired *t*-tests; ns denotes not significant; lines and error bars represent mean and standard deviation, respectively.

Supplementary Table 1 Table showing genotypes of *Drosophila* lines used in this study.
