# Supplementary figures and images for: Macrophage subpopulation identity in Drosophila is modulated by apoptotic cell clearance and related signalling pathways
Source: Front Immunol. 2024 Jan 12;14:1310117. doi: 10.3389/fimmu.2023.1310117 (PMC10811221; doi:10.3389/fimmu.2023.1310117)

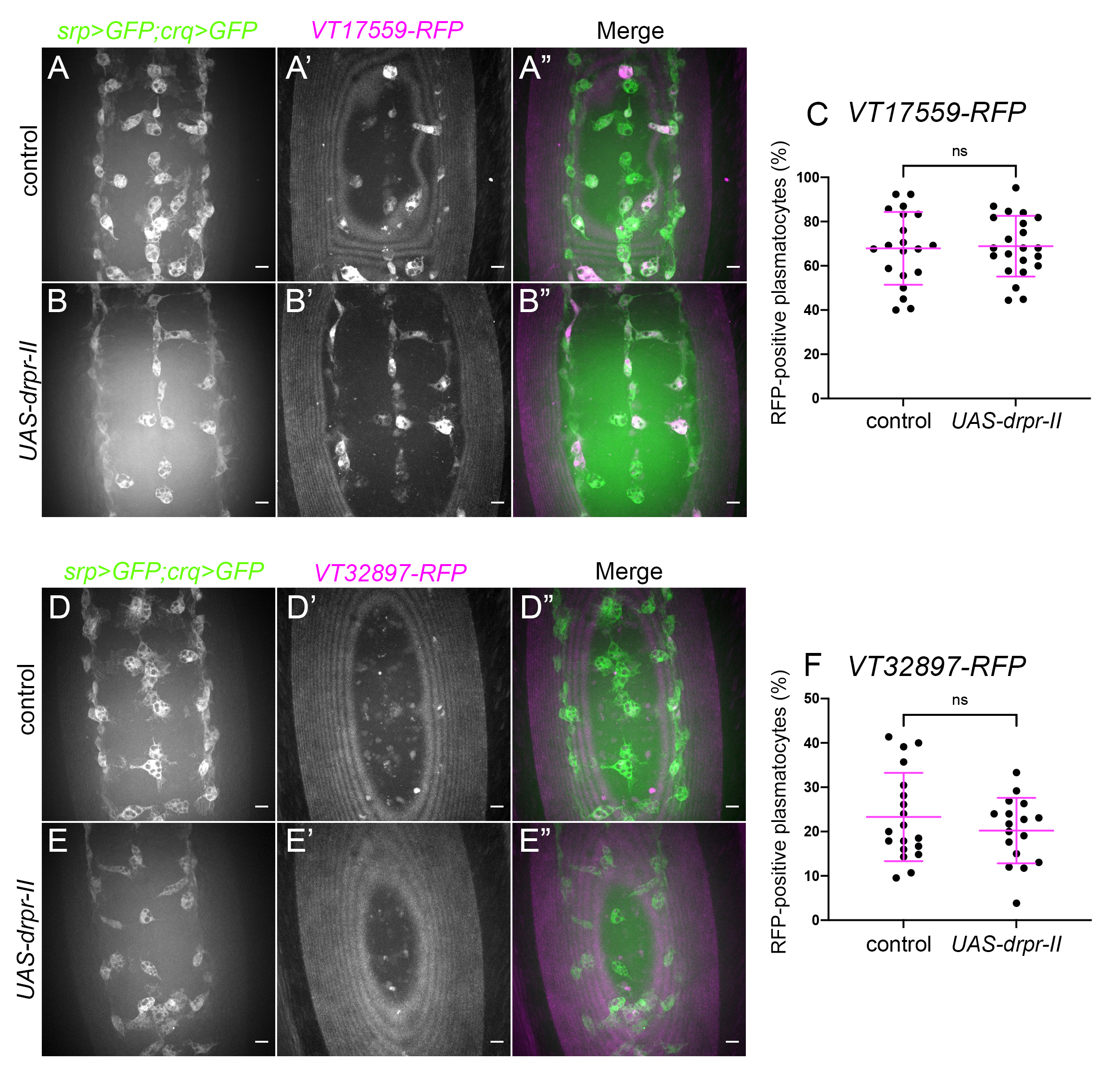

Supplement: Supplementary Figure 1 — Pan-macrophage expression of Draper-II causes no effect on the VT17559 or VT32897 subpopulations. (A-B”, D-E”) representative maximum projection images of the ventral midline of wild-type control embryos (A-A”, D-D”) and embryos expressing UAS-drpr-II specifically in plasmatocytes (B-B”, E-E”) at stage 15. Plasmatocytes labelled via srp-GAL4,UAS-GFP;crq-GAL4,UAS-GFP (A, B, D, E) while subpopulation macrophages labelled via VT17559-RFP (A’, B’) or VT32897-RFP (D’, E’). Anterior is up in all images; scale bars denote 10μm. (C, F) scatterplots showing proportion of plasmatocytes within the VT17559 (C) and VT32897 (F) subpopulations. (C) n= 20 and 22, respectively; (F) n= 19 and 17, respectively. Statistical analyses carried out via unpaired t-tests; ns denotes not significant; lines and error bars represent mean and standard deviation, respectively. [file Image_1.jpeg]

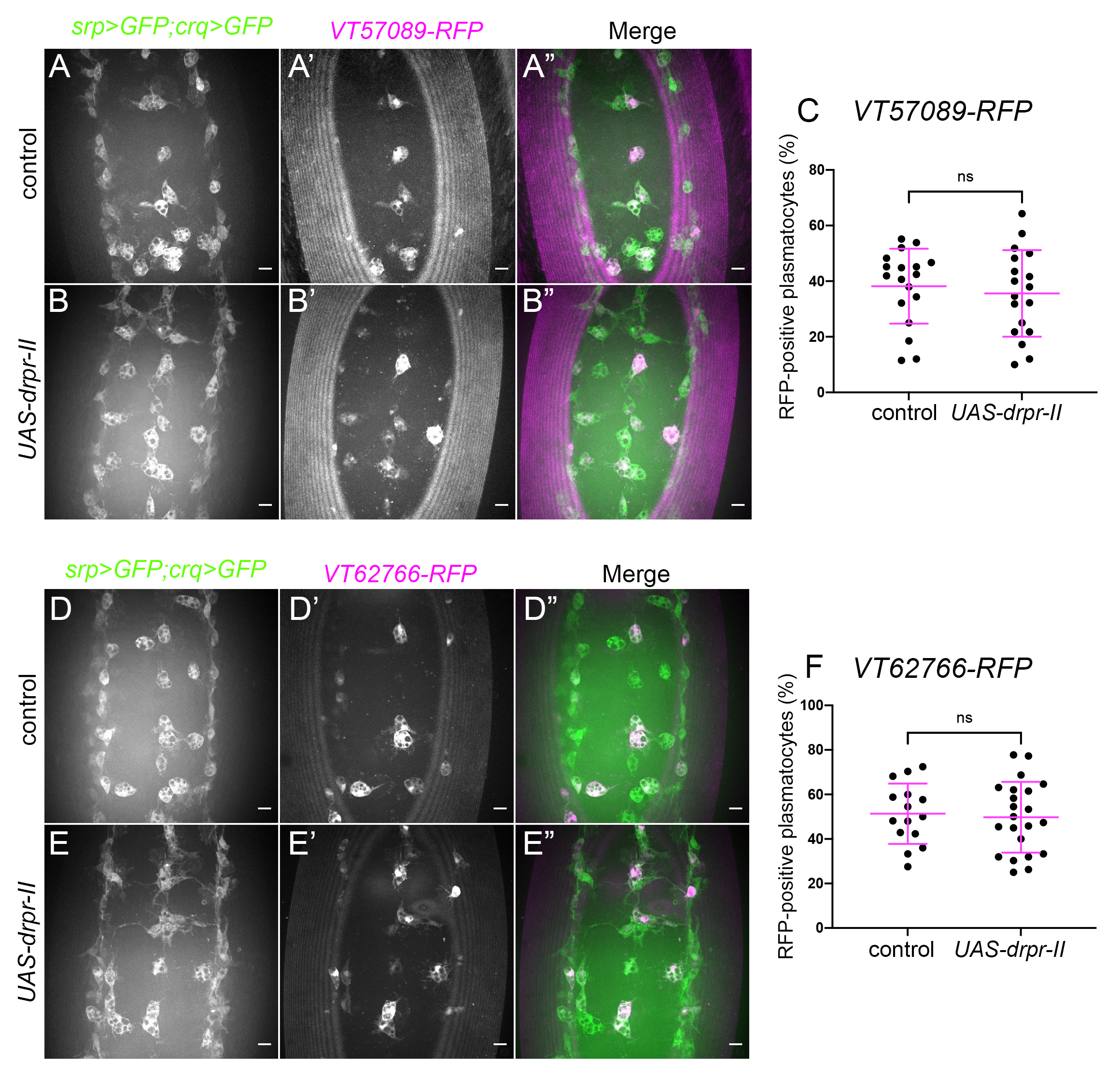

Supplement: Supplementary Figure 2 — Pan-macrophage expression of Draper-II causes no effect on the VT57089 or VT62766 subpopulations. (A-B”, D-E”) representative maximum projection images of the ventral midline of wild-type control embryos (A-A”, D-D”) and embryos expressing UAS-drpr-II specifically in plasmatocytes (B-B”, E-E”) at stage 15. Plasmatocytes labelled via srp-GAL4,UAS-GFP;crq-GAL4,UAS-GFP (A, B, D, E) while subpopulation macrophages labelled via VT57089-RFP (A’, B’) or VT62766-RFP (D’, E’). Anterior is up in all images; scale bars denote 10μm. (C, F) scatterplots showing proportion of plasmatocytes within the VT57089 (C) and VT62766 (F) subpopulations. (C) n= 18 and 18, respectively; (F) n= 15 and 22, respectively. Statistical analyses carried out via unpaired t-tests; ns denotes not significant; lines and error bars represent mean and standard deviation, respectively. [file Image_2.jpeg]
